# Cascara Sagrada in Rheumatism

**Published:** 1888-07

**Authors:** H. T. Goodwin

**Affiliations:** Assistant Surgeon, United States Marine Hospital Service


### *CASCARA SAGRADA IN RHEUMATISM.*

By H. T. GOODWIN, M. D.,
Assistant Surgeon, United States Marine Hospital Service.

The effect of Cascara Sagrada in rheumatism I discovered by accident. About three months ago, I was attacked with severe rheumatic pains in my shoulder, the slightest motion causing intense pain. The third day of the attack I commenced taking as a laxative ten drops of the Cascara, t. i. d. The first morning after taking it the pains were so much less severe that I could move my arm freely. The day following I was entirely free of all discomfort.

Although, as I have intimated, I had not taken the Cascara with any idea of relieving the rheumatism, it occurred to me a few days later that possibly the sudden subsidence of pain might have been due to the drug. There being a few cases of rheumatism in the wards, I determined to try to verify my suspicions. Discontinuing the salicylates, iodides, etc., which these patients were taking, I substituted ext. cascaræ sagradæ fl., 1 c. c., t. i. d. The result astonished me. Within twenty-four hours, there was marked improvement in every case. One case is especially worthy of notice. The patient was a Swedish sailor, who had been admitted three months previously. He suffered intensely, and, although almost everything had been given him from which relief might be expected, his suffering was not allayed. For a day or two after admission, he improved on large doses of salicylate of sodium, but subsequently the pains returned as badly as ever, and the salicylate had no further beneficial effect. Iodide of potassium was given several different times, but, owing to an idiosyncrasy, could be continued only two days at a time, a profuse rash making its appearance over the patient's entire body, the pains remaining as acute as ever. They were not confined to two or three joints, but felt in all, being more severe, however, in the wrists, finger-joints, and ankles, all of which sometimes became œdematous. On the evening of February 5th, I commenced the exhibition of fifteen-drop doses of Cascara Sagrada three times daily. The following morning he was about the same; the second day he was much better; on the seventh he was so far recovered that he asked and obtained permission to walk out. From this on he continued to improve steadily, and on the 17th of February was discharged recovered.

I have since used the Cascara in fully thirty cases, some ten of which were in out-patients, and, with the exception of three or four, in which there was a syphilitic taint, I have obtained the most satisfactory results. I commenced with 1 c. c., t. i. d., and have, so far, never had to increase it beyond 1.5 c. c., and even to this extent in but two cases. I have seldom had to wait beyond twenty-four hours for beneficial effects. In two cases, I had to stop it temporarily, owing to its opening the bowels too freely. In such cases, I would suggest that one of the preparations of iron be given (separately) at the same time. I usually combine it with syrup or glycerin in equal parts, and instruct the patient to take from thirty to forty drops in water. In one case, in which neither it nor the salicylate of sodium appeared to give much benefit, I combined the two with good effect. It is but seldom the bowels are opened too freely by it, the cases above referred to being the only ones I have so far observed.

Among the out-patients upon whom I have used it, were two intelligent officers of vessels. One was an old river pilot, who had periodically suffered intensely for years. I gave him equal parts of the Cascara and syrup, of which I instructed him to take 2 c. c., t. i. d., and requested him to see me again in three days. He returned a month later, and then only to get the medicine renewed. He reported that he had never before had anything relieve him so quickly. The pains began to abate within twenty-four hours after taking the first dose, and in three days after left him entirely. He had had no return, but for fear of another attack, had come to ask for a bottle to keep with him.

The second case was that of Mr. R., first clerk on a large river steamer. He was suffering so much with pain in the hip-joint and thigh, that he could scarcely get to the office. I put him on large doses of salycilate of sodium, with colchicum and iodide of potassium, and instructed him to return in a day or two. In a week, he sent a friend to say that the pain, instead of lessening, was so severe that he could not get to the office. The salicylate, etc., were stopped, and he was given Cascara syrup, thirty-five drops, t. i. d. This was on Friday afternoon. On Sunday, he came to the hospital and reported that he had commenced taking the second prescription Saturday morning, and that on Sunday he had felt decidedly better. He was ordered to continue the drops, and report on Wednesday. Tuesday he sent word that he should be unable to report, as he was sufficiently recovered to resume his usual place on the steamer.

I am not able to explain the action of the drug in relieving rheumatism; I leave that to other observers. I write this in the hope of inducing other medical men to use the Cascara, report their experience, and indicate, more particularly, in what class of cases they have found it of most benefit.

